# Expansion of CRISPR Targeting Sites Using an Integrated Gene-Editing System in *Apis mellifera*

**DOI:** 10.3390/insects12100954

**Published:** 2021-10-19

**Authors:** Liqiang Liang, Zhenghanqing Li, Qiufang Li, Xiuxiu Wang, Songkun Su, Hongyi Nie

**Affiliations:** 1 College of Animal Sciences (College of Bee Science), Fujian Agriculture and Forestry University, Fuzhou 350002, China; mifengwang122@163.com (L.L.); hanqing0320@163.com (Z.L.); lqf2925031791@163.com (Q.L.); xiuwang0393@hotmail.com (X.W.); 2 College of Life Science, Fujian Agriculture and Forestry University, Fuzhou 350002, China

**Keywords:** CRISPR/Cas9, gene editing, *Apis mellifera*, editing scope, NGG

## Abstract

**Simple Summary:**

CRISPR/Cas9, a versatile gene manipulation tool, has been harnessed for targeted genome engineering in honeybees. However, until now, only SpCas9 that enables NGG recognition has been shown to manipulate the genome in *A. mellifera*, limiting the editable range to the NGG-included loci. In the current study, to evaluate the potential expansion when utilising Cpf1, SpCas9 and SaCas9, we predicted the distribution and number of targeting sites throughout the whole honeybee genome with a bioinformatic approach. The results of bioinformatics analysis suggest that the number of accessible targeting sites in *A. mellifera* could be significantly increased via the integrated CRISPR system. In addition, we measured the cleavage activity of these new CRISPR enzymes in *A. mellifera*, and it was found that both SaCas9 and Cpf1 can induce genome alternation in *A. mellifera*, albeit with relatively lower mutagenesis rates for Cpf1 and unstable editing for SaCas9. To our knowledge, our study provides the first evidence that SaCas9 and Cpf1 can efficiently mediate genome sequence mutation, thereby expanding the targetable spectrum in *A. mellifera*. The integrated CRISPR system will probably boost both fundamental studies and applied researches in *A. mellifera* and perhaps other insects.

**Abstract:**

CRISPR/Cas9, a predominant gene-editing tool, has been utilised to dissect the gene function in *Apis mellifera*. However, only the genomic region containing NGG PAM could be recognised and edited in *A. mellifera*, seriously hampering the application of CRISPR technology in honeybees. In this study, we carried out the bioinformatics analysis for genome-wide targeting sites of NGG, TTN, and NNGRRT to determine the potential expansion of the SpCas9, SaCas9, Cpf1, and it was found that the targetable spectrum of the CRISPR editing system could be markedly extended via the integrated gene manipulation system. Meanwhile, the single guide RNA (sgRNA)/crRNA of different novel gene editing systems and the corresponding CRISPR proteins were co-injected into honeybee embryos, and their feasibility was tested in *A. mellifera*. The sequencing data revealed that both SaCas9 and Cpf1 are capable of mediating mutation in *A. mellifera*, albeit with relatively lower mutagenesis rates for Cpf1 and unstable editing for SaCas9. To our knowledge, our results provide the first demonstration that SaCas9 and Cpf1 can function to induce genome sequence alternation, which extended the editing scope to the targets with TTN and NNGRRT and enabled CRISPR-based genome research in a broader range in *A*. *mellifera*.

## 1. Introduction

CRISPR/Cas9 system has dominated the gene-editing field due to its high editing efficiency, universal applicability, and facile implementation. It is widely utilised to conduct gene functional elucidation across a number of insect species, including *Apis mellifera* [1]. Compared with other model insects, research on gene-editing applications in honeybees is relatively rare and only involves the aspects of sex determination, growth and development, antibody-specific verification, and chemical communication. In 2016, Kohno et al. proposed the CRISPR/Cas9 gene-editing method for honeybees, and generated the *major royal jelly protein 1* mutant drone honeybees for the first time [2]. Gene knockout efficiency was considerably improved by changing the injection time and location, as well as the ratio of sgRNA and Cas9 protein/mRNA [3,4]. In 2020, CRISPR/Cas9 technology was applied to chemical communication, and the results showed that gustatory receptor 3 is a highly specific fructose receptor, and *Orco* (*odorant receptor co-receptor*) plays a critical role in the morphology development of the antennal lobe and the expression of genes associated with OR tuning receptors in *A. mellifera* [5,6]. Recently, our group generated a *yellow-y* mutant with dramatic body pigmentation defect, confirming its role during melanin formation in *A. mellifera* [7]. Although CRISPR-mediated gene-editing technology has been established successfully in honeybees, to date, only the SpCas9 system, which specifically recognises NGG protospacer adjacent motif (PAM) sequence, has been applied to targetable genome engineering, thereby constraining the targeting region to the genomic locus containing NGG. The whole-genome sequencing of *A. mellifera* has revealed a high AT content (67%) [8], which increases the difficulty of finding NGG-included loci. Furthermore, a previous study has shown that although NGG target sites can be identified in most genes, there are still 28.39% of genes lacking appropriate targeting sites in *A. mellifera* [9]. To a certain degree, the targetable region limitation hampers the application of CRISPR-Cas-mediated gene editing, such as verifying the function of several special SNP locus and genes without a target of NGG.

To overcome the constraint involved in the PAM requirement of NGG, some Cas9 homologues, derived from other relevant microorganisms and enabling recognition of different types of PAM sequence, were isolated and applied to induce target gene function loss in humans and mammals. For instance, compared with SpCas9, the SaCas9 identified from *Staphylococcus aureus*, harnessed the longer 5′-NNGRRT-3′ (N = A, T, C, or G; R = A or G) as its PAM recognition site and was over 1 kilobase shorter than SpCas9, enabling more convenient package and delivery via viral expression systems [10]. In addition, the Cpf1 protein, isolated and characterised from *Lachnospiraceae bacterium NDND2006* and *Acidaminococcus sp. BV3L6*, selectively recognises a 5′-T-rich sequence (5′-TTTN-3′), which a shorter crRNA directs to cleave targetable genetic areas and is capable to generate double-strand breaks (DSBs) with sticky ends [11,12]. Researchers also developed several artificially modified and engineered Cas9 variants with distinct PAM recognition to reduce the targetable region constraint, including VQR, EQR, and VRER SpCas9 variants, SpCas9-G, ×Cas9, KKH SaCas9, to mediate more precise genetic modifications and broaden the accessible targeting region [13,14,15,16]. These natural Cas9 orthologues and rationally engineered variants strikingly increase the number of accessible targetable sites for gene-editing applications mediated by CRISPR systems, whereas the targeting performance that these CRISPR nucleases induce the genomic modification in *A. mellifera* remains to be investigated.

To test the genome cleavage capability of SpCas9, SaCas9, and Cpf1 in *A. mellifera*, we selected *Amyellow-y* as the target gene, which regulates the biosynthesis of melanin and cannot cause lethality [7]. In the present study, we predicted the numbers of potential NGG, NNGRRT, and TTN sites on every chromosome with bioinformatics method and verified the cleavage activity of SpCas9, SaCas9, and Cpf1 systems in *A. mellifera* embryos. Our results implicated that these CRISPR proteins were functional to efficiently induce gene mutation, albeit with relatively lower mutagenesis rates in some targetable loci, which extended the targeting scope and accelerated the molecular breeding process in *A. mellifera*.

## 2. Materials and Methods

### 2.1. Bioinformatics Analysis of Targeting Sites

On the basis of the high-quality reference genome of *A. mellifera* downloaded from NCBI (https://www.ncbi.nlm.nih.gov/genome/?term=apis+mellifera, accessed on 5 August 2020) [17], the NGG, NNGRRT and TTN sequence patterns were used to determine the SpCas9, SaCas9, and Cpf1 target sites in *A. mellifera*, respectively. The frequencies of NGG, NNGRRT, and TTN editable target sites in *Amyellow-y* mRNA (NM_001098223.1) were evaluated. Then, the number and distribution of the three types of CRISPR target sites on each chromosome were analysed. The average distance between two adjacent targetable sites was also calculated.

### 2.2. Collection of Honeybee Embryos

Honeybee colonies with queens naturally mating within one year were maintained in the apiary of College of Animal Sciences (College of Bee Science), Fujian Agriculture and Forestry University, Fuzhou, China (26.08° N, 119.28° E) in October and November 2019. To encourage the queen to lay more eggs, the colonies were fed with sufficient honey and pollens during the whole experiment process. Therefore, to make queens adapt to oviposition in excluder, queens were confined for 7 consecutive days. For micro-injection, mated queens were limited in the new egg-free comb to obtain time-controlled embryos (within 1.5 h after egg deposition) using plastic queen cages (72 mm × 51 mm × 13 mm) for 1.5 h.

### 2.3. In Vitro Transcription of sgRNA/crRNA and Preparation of CRISPR Protein

sgRNAs of SpCas9 were synthesised according to our previous method [7]. sgRNAs SaCas9 were synthesised according to the kit’s instruction (VK012, Viewsolid, Beijing, China). The crRNAs of Cpf1 were transcribed in vitro using a previous protocol reported by Fernandez [18], and the detailed procedure was as follows: sgRNA/crRNA transcription templates in vitro were produced via a fill-in PCR method with a pair of specific primers. The forward primer encompassed the T7 RNA polymerase-binding sequence and the mature CrRNA preceded by 5′GG. The reverse primer was composed of the target-binding sequence and the repeat sequence. The oligonucleotide annealing was performed with Phanta^®^ Super-Fidelity DNA Polymerase (Vazyme, Nanjing, China) following the parameters: denaturing for 3 min at 95 °C, followed by 30 cycles at 95 °C for 30 s, 52 °C for 30 s and 72 °C for 20 s, and a final elongation step at 72 °C for 7 min. The purified PCR product was cloned into the pEASY-blunt vector (TransGene Biotech, Beijing, China), and subjected to sequencing for confirming the correction of the transcription template sequence. Then, sgRNAs/crRNA were transcripted in vitro using a T7 high yield RNA transcription kit (Vazyme Biotech, Nanjing, China) following the provider’s manual. Reactions were incubated at 37 °C overnight to increase the transcription yield. sgRNAs/crRNA were purified via ethanol precipitation and quantified using Nano-drop 2000 (Thermo Fisher Scientific, Shanghai, China). Then, the purified sgRNAs/crRNAs were run on the 1% agarose gel to ensure its integrality. Cleavage in vitro was performed using targets efficiency detection kit (Viewsolid, Beijing, China) to measure the cleavage activity of sgRNAs/crRNA.

Purified SpCas9 protein (TrueCutTM Cas9 Protein V1) was purchased from Thermo Fisher Scientific (Shanghai, China). EnGen^®^ Sau Cas9 protein (#M0654T) and EnGen^®^ Lba Cpf1 protein (#M0653T) were purchased from NEB (Beijing, China).

### 2.4. Microinjection and Rearing of Embryos

Newly laid embryos were quickly collected and then lined up on the edge of a wax strip using artificially modified moving pipettes. The honeybee embryo micro-injection operation was administered following the approach reported by Schulte [19], with minor modifications. Equal volumes of 1000 ng/μL sgRNA and 1000 ng/μL CRISPR protein were mixed and co-injected into the dorsal posterior region around which germ cells formed using a manual microinjection machine (PLI-100, Medical Systems Corporation). Three hundred embryos were injected per target site. Purified water was injected as a negative control. To reduce the damage to the chorion, the side of the egg should be nearly parallel to the injection pipettes. The entire microinjection process should be accomplished within 1 h to promote the gene knockout frequency. Then, the injected embryos were cultured at 34.5 °C and relative humidity of 90%.

### 2.5. Genomic DNA Isolation and Identification of Amyellow-y Mutations

Briefly, 48 h post-injection, approximately 100 embryos were collected into a 1.5 mL tube and frozen immediately with liquid nitrogen. Honeybee embryo DNA was isolated and purified using the phenol–chloroform extraction and alcohol precipitation method [20]. The amplicon of the intended targeting region was amplified from approximately 70 ng genome DNA template using Max Super-Fidelity DNA Polymerase (Vazyme Biotech, Nanjing, China) with the site-specific primers (Table S1) for *Amyellow-y*. Thereafter, the purification of PCR products was performed with the Omega gel extraction kit (Omega, Guangzhou, China). Cloning was carried out using the pEASY-blunt vector (TransGene Biotech, Beijing, China) to analyse the mutagenesis efficiency and types. Then, 100 individual clones were picked randomly and subsequently sent to Biosune Biotechnology Company (Fuzhou, China) for Sanger sequencing. For several target sites where any mutation was not detected via Sanger sequencing, the deep sequencing of site-included PCR amplicons was conducted on an Illumina MiSeq platform (Sangon Biotech, Shanghai, China). The number of reads for the deep sequencing was ~40,000. Sequence alignments were carried out with BioEdit8.1.0 software.

## 3. Results

### 3.1. Comparative Analysis of Targetable Sites on Amyellow-y mRNA

**Figure 1:**
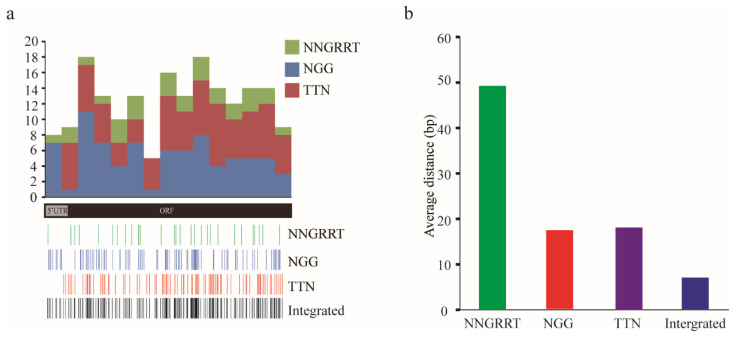
Comparative analysis of CRISPR targeting sites on *Amyellow-y* mRNA: (**a**) numbers and distributions of targetable sites on *Amyellow-y* mRNA. The different colours of the top panel indicate the number of different CRISPR target sites. The *X*-axis indicates the position on *Amyellow-y* mRNA, and the *Y*-axis represents the numbers of targeted sites. The middle panel presents the schematic diagram of the *Amyellow-y* mRNA structure. The Bottom panel is the sketch map of sites distribution. Each line indicates one target site; (**b**) the average distance between two adjacent sites of different CRISPR systems along *Amyellow-y* mRNA. The *X*-axis represents the PAM types, and the *Y*-axis represents the average distances.

To evaluate the potential expansion when utilising the SpCas9, SaCas9, Cpf1 in *A. mellifera*, we first adopted *Amyellow-y* mRNA as an example and analysed the number of NGG, NNGRRT, and TTN target sites. Only 157 NGG sites were identified on *Amyellow-y* mRNA when only utilising SpCas9. However, a total of 505 sites including 157 NGG, 73 NNGRRT, and 275 TTN were found, which showed a 2.2-fold increase of targetable sites number when combining SpCas9, SaCas9, and Cpf1 systems. Most regions were covered by sites in the integrated CRISPR system (Figure 1a). The average distances between two adjacent sites of NGG, NNGRRT, and TTN were 18.0 bp, 49.5 bp, and 18.6 bp, respectively. Additionally, the integrated CRISPR system expanded the targeted region to 7.7 bp along *Amyellow-y* mRNA (Figure 1b).

### 3.2. Bioinformatics Analysis of Targetable Sites on A. mellifera Genome

**Figure 2:**
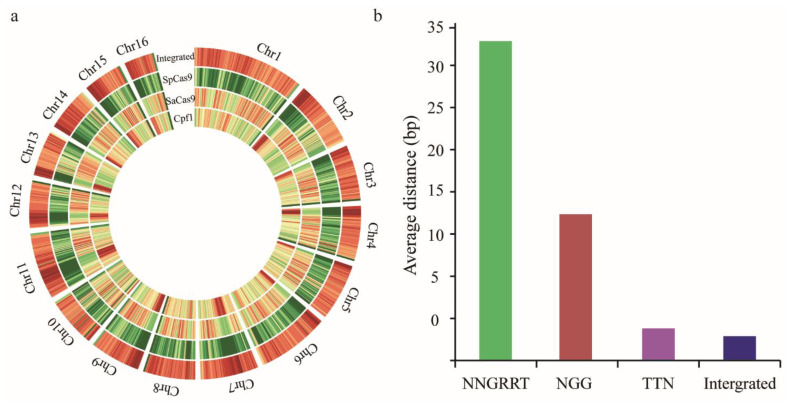
Bioinformatics analysis of CRISPR targeting sites on *A. mellifera* chromosome: (**a**) distribution of different target sites’ position on a chromosome. The red and green colour indicates high density and low density of CRISPR sites, respectively; (**b**) the average distance of two adjacent sites along the whole chromosome.

**Figure 3:**
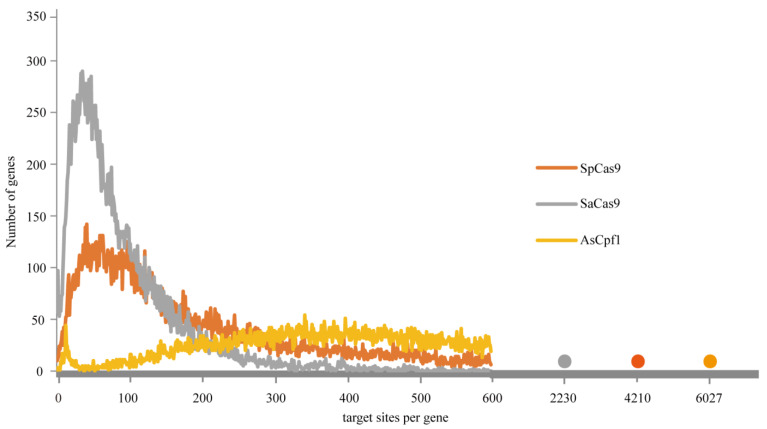
The number of CRISPR targeting sites corresponding to gene numbers.

Genome-wide analysis using the bioinformatics approach was conducted to predict all potential targetable sites on every chromosome of *A. mellifera*. A total of 77,599,237 sites (12,878,811 NGG, 5,894,281 NNGRRT and 58,826,145 TTN sites) were identified. A 5.0-fold increase in the number of targetable sites was achieved when utilising the integrated CRISPR system than when only using SpCas9. For the integrated genome-editing platform, the largest number of CRISPR sites were on chromosome 1, but the least number of CRISPR sites were on chromosome 16 (Table S2). Whole-genome distribution analysis indicated that most of the targeting sites were relatively uniformly distributed on the honeybee chromosome (Figure 2a). The average distances of two adjacent targeting sites and genome-wide targeting frequency were also analysed. The mean distances for SpCas9, SaCas9, and Cpf1 are 17.3 bp, 37.7 bp, and 3.8 bp, respectively. The frequency of Cpf1 target sites was the highest through the whole genome, followed by SpCas9, and that of SaCas9 was the lowest. When using the integrated gene manipulated tools, the site distances could be decreased to 2.9 bp (Figure 2b). Moreover, we calculated the number of target sites of NGG, NNGRRT, and TTN per gene. TTN was the most frequent (1–6027 sites per gene), and the majority of genes contain 100 to 1000 sites. NNGRRT was the least frequent (0–2230 sites per gene), and most genes contain fewer than 100 sites. A great number of genes include 50 to 200 targeted sites of the NGG pattern (Figure 3). Collectively, the targetable spectrum was markedly extended using the integrated platform, enabling more intended genome locations accessible to be edited.

### 3.3. Genome Editing Mediated by SpCas9, SaCas9 and Cpf1 in A. mellifera

**Figure 4:**
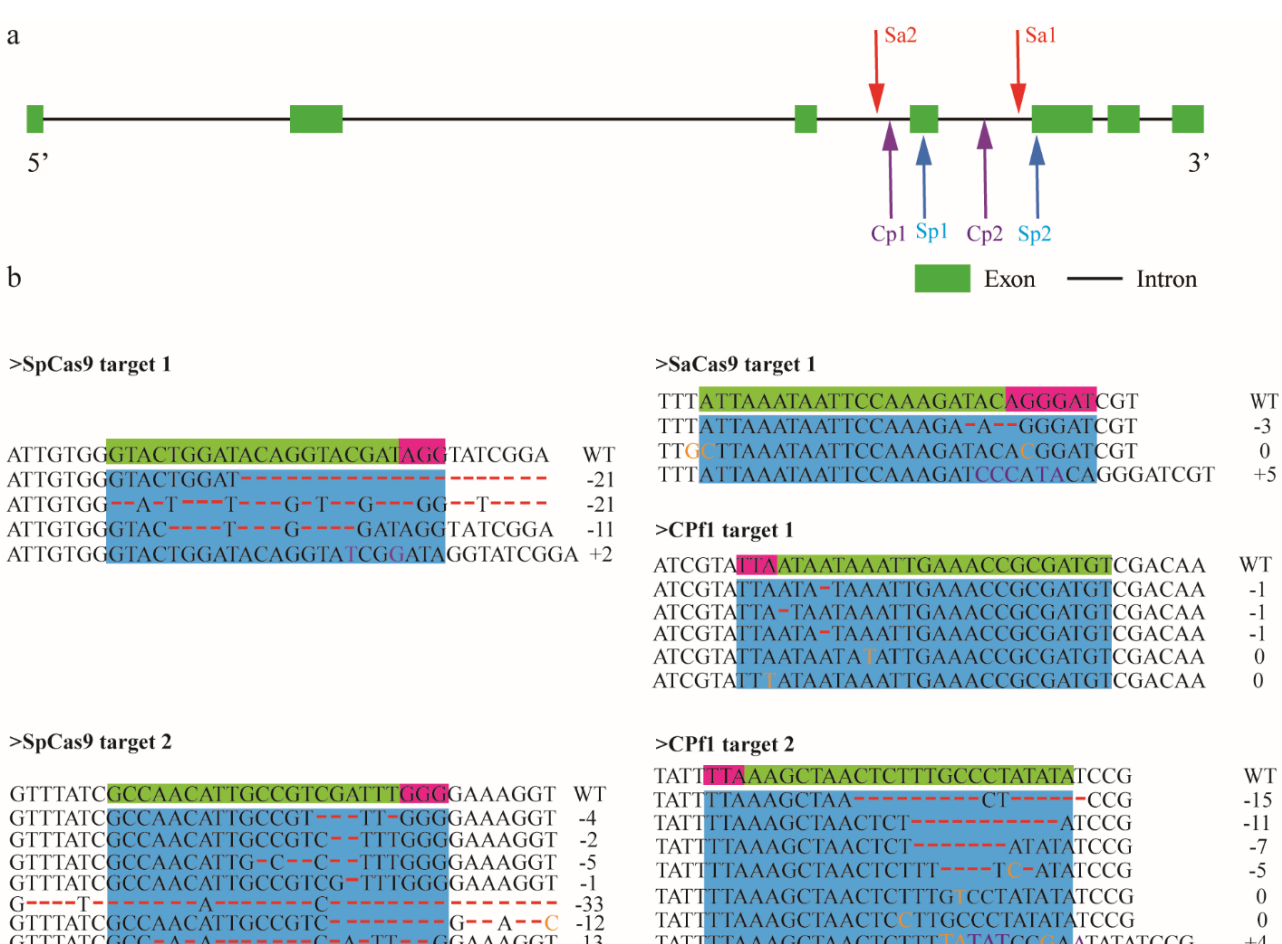
Gene mutation mediated by SpCas9, SaCas9, and Cpf1 in *A. mellifera* embryos: (**a**) schematic diagram of gene structure and target sites position on *Amyellow-y* gene. The green rectangle and black line represent exons and introns, respectively. The sgRNA site position is indicated by chromatic arrows. Sa1 and Sa2 represent target 1 and target 2 of SaCas9, respectively. Cp1 and Cp2 indicate target 1 and target 2 of Cpf1, respectively. Target 1 and target 2 of SpCas9 are presented as Sp1 and Sp2, respectively; (**b**) *Amyellow-y* mutation patterns induced by different CRISPR systems. The left panel indicates mutation patterns introduced by Spcas9. The top figure of the right panel represents mutation patterns mediated by Sacas9. The middle figure and the bottom figure of the right panel indicate mutation patterns induced by Cpf1. PAM motif and targetable site are presented as letters in pink and light green boxes, respectively. The insertion events are lettered in purple, the substituted bases were denoted by orange letters, and the base deletion is represented by red dashed lines. The spacer sequence and PAM motif are highlighted in blue colour. The black number labelled at the right of each mutation sequence represents altered nucleotide length (−, deletion; +, insertion; 0, substitution). WT indicates the honeybee embryos injected with purified water.

To investigate the capability that SpCas9, SaCas9, and Cpf1 introduce target mutagenesis, we designed two sgRNAs corresponding to every CRISPR system to knock out *Amyellow-y* gene (Figure 4a). Since the base mutation of the seed sequence can prevent the cleavage activity of the Cas9 enzyme [21], the genome amplicons containing all the target sites we designed were amplified and sequenced to avoid the SNP presence on targetable sites. The sequencing results suggested that the sequenced fragments were identical to the sequence loaded from the NCBI database, and no obvious SNP was found. The mutation events were verified via TA-clone sequencing and deep sequencing (Figure 4b). According to the Sanger sequencing results, substitution and indels (insertions and deletions) took place in the vicinity of target sites for SpCas9-mediated mutations. The detected mutation rates of the two target sites were 10% and 16%, respectively. For SaCas9, TA clone results showed the high-efficiency and versatile mutations occur around target 1, with a frequency of 71%. However, any mutation in target 2 was not detected using both Sanger sequencing and next-generation sequencing. For Cpf1, the mutagenesis events in neither of the two were found utilising TA clone sequencing. Additionally, deep-sequencing results revealed that mutations of target 1 and target 2 were 0.7% and 14%, respectively. Notable differences were observed in mutation frequency for alternative sgRNAs of SaCas9 and Cpf1, possibly because sgRNAs form different secondary structures, which influences the binding ability between sgRNA and genome [22]. Our results indicated that SaCas9 and Cpf1 enzymes can mediate sequence mutation in *A. mellifera*, albeit with the lower cleavage frequency of the Cpf1 enzyme and the weaker stability of SaCas9 in inducing mutagenesis, compared with SpCas9.

## 4. Discussion

One of the critical determinants for successfully carrying out CRISPR-based experiments is to ensure as high cleavage frequency as possible [23,24,25]. Our sequencing results showed that the gene knockout efficiencies of SpCas9, SaCas9, and Cpf1 in *A. mellifera* were 10~16%, 0~71%, and 0.7~14%, respectively, which is in accordance with the previous result that Cpf1 exhibited considerably lower cleavage activity in comparison with SpCas9 in *Drosophila melanogaster* [26]. During the past few years, a series of effective approaches have been adopted to increase gene knockout efficiency. Dang et al. demonstrated that modifying and optimising the structure of sgRNA strikingly promoted cleavage activity in SpCas9-based research [27]. Liu et al. obtained an obvious efficiency increase from 4% to 39% via constructing a zebrafish-codon-optimised SpCas9 [28]. By changing the proportion and concentration of Cas9 and sgRNA, Roth et al. further increased the mutation rate to 100% and obtained the entirely mutated worker bees directly without producing mutant queens and drones [3]. Furthermore, Hu et al. established a higher-efficiency CRISPR/Cas9 method in *A. mellifera* embryos by adjusting the delivery method of Cas9/sgRNA, injection time, and position [4]. Bassett et al. and Li et al. suggested that increasing the injection concentration of CRISPR protein/mRNA and sgRNA can promote knockout efficiency [29,30]. Similarly, these strategies, which have been successfully used to promote SpCas9 editing capability, can also be utilised to improve the targeting activity of Cpf1 and SaCas9 in *A. mellifera*.

Editing range is also of paramount significance to induce gene sequence changes precisely and dissect gene function [23]. Similar to humans and mammals, to address the SpCas9 target site limitation, several artificially engineered SpCas9 derivatives and novel CRISPR proteins homologous to SpCas9, which enable distinct PAM recognition, have been utilised to generate DSBs and interrogate gene function in several model insects, such as *Drosophila melanogaster* and *Bombyx mori*. Ma et al. inserted codon-optimised SaCas9 and Cpf1 coded sequences into the expression vector and transfected into silkworm BmN-SWU1 cell lines, demonstrating that both SaCas9 and Cpf1 systems can successfully introduce mutagenesis and their mutation efficiency is equivalent to that of SpCas9 [23]. Ni et al. employed the CRISPR/×Cas9 system to induce DSBs in the target gene *white* and compared it with the cleaving activity of different target sites in *D. melanogaster*; their results suggested that ×Cas9 manifested efficient cleavage on GGG and TGA target sites [31]. Port et al. injected the plasmid expressing Cpf1 and crRNA into *Drosophila melanogaster* embryos to target *e* and successfully broadened the targeting region, albeit with relatively lower mutation frequencies in comparison with SpCas9 [26]. Our study predicted the targeting sites of SpCas9, SaCas9, and Cpf1 on the genome of *A. mellifera* and investigated their editing activity to induce DSBs and function disruption in honeybee for the first time. These results suggested that SaCas9 and Cpf1 are capable to mediate gene sequence changes, which complemented the CRISPR toolbox and enabled genome modification in a wider range in *A. mellifera*.

Potential off-target mutation is a major concern in CRISPR-based gene manipulation experiments [24]. CRISPR/Cas9 system is slightly tolerant to mismatches, usually leading to unintentional site mutation [32]. With the brick development and application of the CRISPR system in the fields of basic researches and applicable studies, the potential off-target effects have caused a major concern of CRISPR-based technology, especially in the preclinical gene treatments of human diseases and gene drive to control invasive species [23,33]. To date, off-target mutagenesis has never been systematically and unbiasedly estimated in *A. mellifera*, and the off-target mutation cannot still be neglected. One potential approach to address the off-target issue is to utilise several novel CRISPR enzymes that possess weaker off-target effects. Kim et al. and Kleinstiver et al. demonstrated that Cpf1 had enhanced targeting specificity in human cells using a genome-wide approach, with no mutations occurring in most off-target loci [34,35]. According to a previous study, the PAM sequence exerts an obvious effect on the recognition specificity of RNA-guided CRISPR nucleases [36]. SaCas9 utilises a more complicated and stringent PAM sequence in comparison with SpCas9, which may be beneficial to reduce off-target activity, especially in the genome-conserved species. This study is the first report to introduce DSBs utilising SaCas9 and Cpf1 in *A. mellifera*, and the integrated genome-editing system provides a guarantee for the specific knockout. Additionally, truncated 18 nt guide RNAs and several engineered SpCas9 variants with more special PAM affinity have been reported to have great potential to improve the specialty and reduce off-target effects of CRISPR-based gene abortion [37,38,39,40,41]. In future research, these methods can be used to achieve a more robust specificity of CRISPR experiments in *A. mellifera*.

## 5. Conclusions

We predicted the frequency and distribution feature of targetable sites of SpCas9, SaCas9, and Cpf1 on the honeybee genome and tested their editing performance in *A. mellifera.* Our results indicated that these novel CRISPR systems were functional to edit the genome of *A. mellifera* and notably increased the targetable region. This study provides the first demonstration that introduces gene function loss utilising SaCas9 and Cpf1 in *A. mellifera*. These results provide options for genome modification in *A. mellifera*.

## Data Availability

The data presented in this study are available on request from the first author.

## Supplementary Materials

The following are available online at www.mdpi.com/article/10.3390/insects12100954/s1, Table S1: Primer information used in this study, Table S2: Frequency of CRISPR targeting sites in all 16 honeybee chromosomes.
