# Supplementary material for: Expansion of CRISPR Targeting Sites Using an Integrated Gene-Editing System in Apis mellifera
Source: Insects. 2021 Oct 19;12(10):954. doi: 10.3390/insects12100954 (PMC8540347; doi:10.3390/insects12100954)
Supplement: Supplementary file 1 [file insects-12-00954-s001.zip › insects-1368639-supplementary.pdf]

Table S1 Information of primers used in this study

Primers name	Primer sequence (5' to 3')	Primer purpose
Template-SpCas9 target1-F	TTAATACGACTCACTATAGGGGTACTGGATACAGGTACGATGTTTTAGAGCTAGAAATAG	Amplifying transcription template of sgRNA
Template-SpCas9 target2-F	TTAATACGACTCACTATAGGGGCCAACATTGCCGTCGATTTGTTTTAGAGCTAGAAATAG	Amplifying transcription template of sgRNA
Template-SpCas9 -R	AGCACCGACTCGGTGCCACTTTTTCAAGTTGATAACGGACTAGCCTTATTTTAACTTGCTATTTCTAGCTCTAAA	Amplifying transcription template of sgRNA
Template-SaCas9 target1-F	TAATACGACTCACTATAGATTAAATAATTCCAAAGATACGTTTTAGTACTCTGGAAACAG	Amplifying transcription template of sgRNA
Template-SaCas9 target2-F	TAATACGACTCACTATAGCAAATATTAATGAGACGAGATGTTTTAGTACTCTGGAAACAG	Amplifying transcription template of sgRNA
Template-Cpf1 target12-F	CCCTAATACGACTCACTATAGGTAATTTCTACTAAGTGTAGAT	Amplifying transcription template of CrRNA
Template-Cpf1 target1-R	ACATCGCGGTTTCAATTTATTATATCTACACTTAGTAGAAATTA	Amplifying transcription template of CrRNA
Template-Cpf1 target2-R	TATATAGGGCAAAGAGTTAGCTTATCTACACTTAGTAGAAATTA	Amplifying transcription template of CrRNA
Mu-SpCas9 target1-F	AGTTGGTTCGTTCTCGCTTGAATAATG	Detecting mutations
Mu-SpCas9 target1-R	TGTCTTCGGCCCTCAGTC	Detecting mutations
Mu-SpCas9 target2-F	GAATGTAAGAAAGAACACCGAAC	Detecting mutations
Mu-SpCas9 target2-R	CTGCTCCCACGAGTAAACG	Detecting mutations
Mu-SaCas9 target1-F	AGTTGGTTCGTTCTCGCTTGAATAATG	Detecting mutations
Mu-SaCas9 target1-R	ATCTGTTCTGCTCCCACGAGTAAAC	Detecting mutations
Mu-SaCas9 target2-F	CAGTTGGTTCGTTCTCGCTTGAATAATG	Detecting mutations
Mu-SaCas9 target2-R	CGCTGGTCAGGTCGAAGATGTTG	Detecting mutations
Mu- Cpf1 target1-F	CAGTTGGTTCGTTCTCGCTTGAATAATG	Detecting mutations
Mu- Cpf1 target1-R	CGCTGGTCAGGTCGAAGATGTTG	Detecting mutations
Mu- Cpf1 target2-F	CATCTCGGTGGTAGTCGCACTC	Detecting mutations
Mu- Cpf1 target2-R	CGACGGCAATGTTGGCGATAAAC	Detecting mutations

Table S2 Frequency of CRISPR targeting sites in all honeybee 16 chromosomes

chr.	Length	TTN	NGG	NNGRRT	The integrated system
**chr1**	27754200	7111456	1703894	753122	9568472
**chr2**	16093500	4091992	1031086	426048	5549126
**chr3**	13619445	3613676	783131	369438	4766245
**chr4**	13404451	3512073	810385	357949	4680407
**chr5**	13920984	3616030	846742	376499	4839271
**chr6**	17812983	4766166	977883	470703	6214752
**chr7**	14262382	4052546	684171	363842	5100559
**chr8**	12786769	3456449	719831	346378	4522658
**chr9**	12729961	3512008	678441	334110	4524559
**chr10**	12657134	3045754	773391	320922	4140067
**chr11**	16444887	4551683	829267	415422	5796372
**chr12**	11533137	3033628	693013	312725	4039366
**chr13**	11279722	2872073	749752	306298	3928123
**chr14**	10910039	2921990	649760	293352	3865102
**chr15**	9922473	2691121	561361	260608	3513090
**chr16**	7238532	1977500	386703	186865	2551068
**Totality**	222370599	58826145	12878811	5894281	77599237

Numbers in purple and red represents the total length of chromosome and the total number of targeting sites, respectively.
